# Genetic parameters for pelvic organ prolapse in purebred and crossbred sows

**DOI:** 10.3389/fgene.2024.1441303

**Published:** 2024-07-31

**Authors:** Ching-Yi Chen, Pieter W. Knap, Adria S. Bhatnagar, Shogo Tsuruta, Daniela Lourenco, Ignacy Misztal, Justin W. Holl

**Affiliations:** ^1^ The Pig Improvement Company, Genus plc, Hendersonville, TN, United States; ^2^ The Pig Improvement Company, Genus plc, Isernhagen, Germany; ^3^ Department of Animal and Dairy Science, University of Georgia, Athens, GA, United States

**Keywords:** pigs, sow mortality, vaginal prolapse, uterine prolapse, rectal prolapse, genetic parameters

## Abstract

This study aimed to investigate genetic parameters for sow pelvic organ prolapse in purebred and crossbred herds. Pelvic organ prolapse was recorded as normal or prolapsed on the individual sow level across 32 purebred and 8 crossbred farms. In total, 75,162 purebred Landrace sows from a single maternal line were recorded between 2018 and 2023, while 18,988 commercial two-way crossbred (Landrace x Large White) sows were available between 2020 and 2023. There were 5,122,005 animals included in the pedigree. The prolapse in purebreds and crossbreds was considered two different traits in the model. Pedigrees of the crossbred sows were determined based on genotypes through parentage assignment. The average incidence rates were 1.81% and 3.93% for purebreds and crossbreds, respectively. The bivariate model incorporated fixed effects of parity group and region with random effects of contemporary group (farm and mating year and month at the first parity), additive genetic, and residual. Genetic parameter estimates were obtained using BLUPF90+ with the AIREML option. The estimated additive variance was larger in crossbreds than in purebreds. Estimates of heritability in the observed scale were 0.09 (0.006) for purebreds and 0.11 (0.014) for crossbreds, with a genetic correlation of 0.83 using a linear model. Results suggested that including data from crossbreds with higher incidence rate is beneficial and selection to reduce the prolapse incidence in purebred sow herds would also benefit commercial crossbred sow herds.

## 1 Introduction

Sow lifetime productivity is an important factor of the overall productivity and profitability of the herd. High sow mortality rates lead to the loss of breeding sows, which directly results in a reduced number of piglets born and increased production expenses for sow replacement. Although distinguishing among various removal reasons can be challenging, they can generally be grouped into categories of reproductive, nonreproductive (i.e., physical and health problems), and miscellaneous (Friendship et al., 1986; Arango et al., 2005; Engblom et al., 2007; Mote et al., 2009; Ketchem et al., 2017). Reproductive reasons are often the major cause of sow removal; for example, in the study by Arango et al. (2005), the proportions of the three above categories were 58.4%, 29.3%, and 12.3%, respectively. Regarding removals, events that are recorded as deaths indicate contributing factors to sow mortality. Among the causes of sow mortality, pelvic organ prolapses (e.g., vaginal, uterine, or rectal) have been reported with an increased incidence in the USA since 2013. Pittman (2017) reported the annual average removal reason due to prolapse as 2% from 2008 to 2013, with an increase to 3.5% from 2013 to 2016, and to the extreme case of having farms with 25%–50% sow mortality caused by prolapse. Seasonal variation in the incidence rate was also observed, with the highest incidence when farrowing from January to March and the lowest from June to September. Ross (2019) reported that prolapse accounted for 21% of the total sow mortality in a survey conducted at 104 farms from 15 U.S. states. The average annualized sow mortality due to all causes was 12.7%, with annualized prolapse mortality of 2.7% (from 0.3% to 10.3%) and annualized non-prolapse mortality of 10.0% (from 3.4% to 21.4%).

In pig breeding systems, purebred sows are kept in genetic nucleus farms. In contrast, crossbred sows, benefiting from heterosis, are kept in commercial operations with different environments and health conditions compared to genetic nucleus farms. To expedite genetic progress, purebred sows in genetic nucleus systems are frequently culled before reaching their full lifetime productivity potential, resulting in fewer average parities compared to crossbred sows in commercial operations, which are managed to maximize sow lifetime productivity. The primary goal of genetic selection of elite purebred candidates is to improve the commercial crossbred performance. Differences in genetic make-up and animal management between purebred and crossbred herds may lead to a genetic correlation that is not unity, suggesting a potential for genotype by environment interaction. Many studies (Lutaaya et al., 2001; Bloemhof et al., 2012; Dufrasne et al., 2013; Abell et al., 2016; Fragomeni et al., 2016; Wientjes and Calus, 2017; Christensen et al., 2019; Pocrnic et al., 2019; Steyn et al., 2021) have focused on the combination of purebred and crossbred components, either investigating various research topics (dominance effect, genomic prediction accuracy, and heat stress) or estimating the purebred-crossbred genetic correlation for various traits (growth rate, backfat thickness, carcass traits, birth weight, preweaning mortality, and sow longevity).

Currently, there is less literature available on the genetic parameters of prolapse. Moreover, most of these studies only have data on purebred sows collected from a small number of farms located in the USA (Supakorn et al., 2019; Stevens et al., 2021; Dunkelberger et al., 2022; Bhatia et al., 2023). Acquiring large datasets from purebred and crossbred sows across different environments is crucial not only for a deeper understanding of the genetics of prolapse but also for enhancing accuracy through combined selection, as crossbred data better reflects the condition of interest. This study aims to estimate the genetic parameters for sow pelvic organ prolapse in purebred and crossbred sow herds.

## 2 Materials and methods

### 2.1 Data

Data were provided by PIC (a Genus company, Hendersonville TN, United States). To maximize the variation in environmental conditions, data from North American (NAM) and South American (SAM) regions were analyzed. For NAM, the farms were located in Canada and the USA. For SAM, the farms were located in Brazil. The initial data set contained 341,011 mating records of 130,474 purebred Landrace and crossbred (Landrace x Large White) sows from multiple parities across 58 farms in NAM and SAM. Data for purebred sows were available from 2018 to 2023, and data for crossbred sows were available from 2020 to 2023, respectively. The average parity numbers were 2.37 ± 1.37 and 3.07 ± 1.46 for purebred and crossbred sows, respectively. In this study, sow pelvic organ prolapse was categorized as a binary trait with vaginal, uterine, and rectal prolapses combined into one unified event and analyzed as a single trait. Sows that did not prolapse had the opportunity to progress to the next parity, whereas prolapsed sows were removed from the herd. We opted to use records collected from sows at their early reproductive cycle to identify early indicators for prolapse incidence. Instead of using repeated records across parities per sow, we defined the prolapse event collected up to parity 2, resulting in a single record per sow. The binary trait was assigned as failure if the sow experienced a prolapse and pass otherwise. A prolapse record at parity 1 is recorded for sows currently in production at their first parity, although these data might be subject to censoring. A prolapse record at parity 2 is recorded for sows that have reached their second parity and beyond.

After quality control, only farms with more than 500 sows remained in the analysis. In total, 75,162 purebred sows from a single maternal line on 32 purebred farms and 18,988 commercial two-way crossbred sows on 8 crossbred farms were analyzed. There were 5,122,005 animals included in the pedigree. To establish a connection between the crossbred and their purebred ancestors, the crossbred sows were genotyped, and their pedigree was determined through parentage assignment. Prolapse in purebreds and crossbreds was considered two different traits in the model. - Number of animals used in the analysis and the descriptive statistics are in Table 1.

**TABLE 1:** Number of animals with prolapse recorded and the descriptive statistics.

Item		NAM		SAM		Total
		Parity 1	Parity 2	Parity 1	Parity 2	
Purebred	N	8,463	13,998	17,948	34,753	75,162
	N of prolapsed	189	378	352	440	1,359
	Incidence rate, %	2.23	2.70	1.96	1.27	1.81
	SD, %	14.78	16.21	13.87	11.18	13.33
Crossbred	N	3,769	15,219	0	0	18,988
	N of prolapsed	317	430	0	0	747
	Incidence rate, %	8.41	2.83	-	-	3.93
	SD, %	27.76	16.57	-	-	19.44

NAM, North American region; SAM, South American region.

### 2.2 Statistical analysis

Linear models generally yield estimated breeding values that are strongly correlated with those from threshold models but are computationally less complex (Hidalgo et al., 2024). This allows for easier implementation, making them a practical choice. Genetic parameters were estimated using BLUPF90+ with AIREML option (Lourenco et al., 2022). Analyses were performed using a two-trait linear model:

y=Xβ+Zu+Wc+e,

where y is the vector of phenotypes (purebred or crossbred); β is the vector of fixed effects of region (NAM, SAM) and parity group (1, 2); c is the vector of random contemporary group effects defined as farm and mating year and month at the first parity; u is the vector of random additive genetic effects; e is the vector of random residual effects; **X**, **Z**, and W are the incidence matrices relating prolapse records in vector **y** to effects in β , u , and c , respectively. Random effects were assumed to follow a multivariate normal distribution with a mean of zero and covariance structure as outlined below:

$$Var\left[\begin{array}{c} u\\ c\\ e \end{array}\right]=\left[\begin{array}{ccc} A⊗G & 0 & 0\\ 0 & I⊗C & 0\\ 0 & 0 & I⊗R \end{array}\right],$$

where **A** is the numerator relationship matrix; I is the identity matrix; **G** is the (co)variance matrix of random additive genetic effects; **C** is the (co)variance matrix of random contemporary group effects; R is the residual (co)variance matrix.

## 3 Results

### 3.1 Observed prolapse incidence rate

**FIGURE 1:**
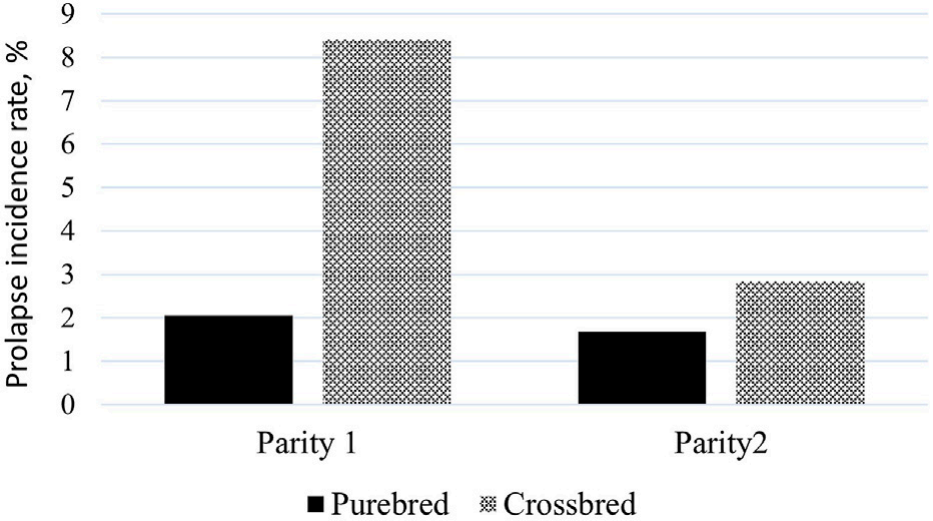
Average prolapse rate within parity for purebred and crossbred sows.

**FIGURE 2:**
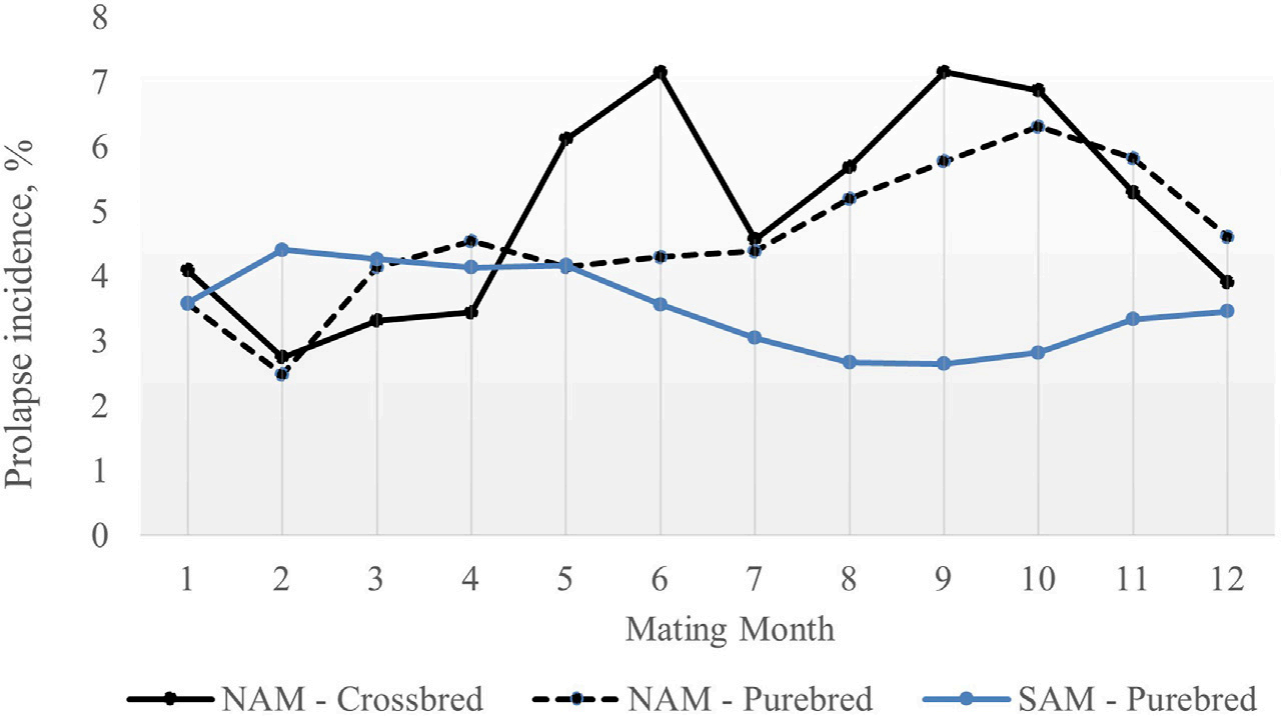
Average prolapse rate by mating month for purebred and crossbred sows NAM: North American region; SAM: South American region. The average prolaspe rate by month is based on data from 2022 January to 2023 January, using a moving average calculated over every two months.

The average prolapse incidence rate was lower in purebred sows compared to crossbred sows across parities, with rates of 1.81% and 3.93%, respectively (Table 1). By parity, the incidence rate was 0.72% in parity 1% and 1.09% in parity 2 for purebred sows, and 1.67% in parity 1% and 2.26% in parity 2 for crossbred sows. Within parity (Figure 1), the incidence rate was 2.05% in parity 1% and 1.68% in parity 2 for purebred sows, and 8.41% in parity 1% and 2.83% in parity 2 for crossbred sows. The seasonal variation for prolapse incidence is shown in Figure 2. Since crossbred data was not available prior to 2020 and prolapse incidence in SAM was near zero before 2021, the dataset from 2022 to 2023 was used to describe the seasonal variations within a single year. The average incidence rate by month is based on data from 2022 January to 2023 January, using a moving average calculated over every 2 months. In NAM, high incidence rates occurred when sows were mated from August to November for both purebred and crossbred sows, with low rates in February. However, there is a peak of incidence rate observed in this data set for purebred sows in May and June. For SAM, higher incidence rates from February to May and lower rates from August to October.

### 3.2 Genetic parameters

Estimates of variance components, heritability, and genetic correlation, in the observed scale, are in Table 2. Estimates of heritability were 0.09 for purebreds and 0.11 for crossbreds. The additive variance (×1000) in crossbreds was 4.18, nearly three times as large as the variance in purebreds (1.51). The residual variance (×1000) in crossbreds was 32.8, approximately twice as large as the variance in purebreds (16.0). The purebred-crossbred genetic correlation was estimated as 0.83, with a standard error of 0.13.

**TABLE 2:** Estimates of genetic parameters (SE) for prolapse incidence.

	Purebred	Crossbred
$\sigma_{a}^{2}$	1.51 (0.112)	4.18 (0.543)
$\sigma_{c}^{2}$	0.16 (0.021)	0.32 (0.077)
$\sigma_{e}^{2}$	16.03 (0.113)	32.77 (0.556)
$h^{2}$	0.09 (0.006)	0.11 (0.014)
$\sigma_{a12}$	2.09 (0.371)	
$r_{g}$	0.83 (0.133)	

$\sigma_{a}^{2},\sigma_{c}^{2},\sigma_{e}^{2}$ : additive genetic, contemporary group, and residual variances; h^2^: heritability; σ_a12_, r_g_: genetic covariance and correlation between purebred and crossbred incidence. Standard errors in parentheses. The (co)variance values shown here are 1000 × the estimates.

## 4 Discussion

Acquiring useful data from crossbred sows is often challenging due to the lack of pedigree information. However, it is essential to incorporate performance data from crossbred sows for commercial relevance, leading to more precise breeding value estimations for both purebreds and crossbreds. This study is the first to estimate genetic parameters of sow pelvic organ prolapse using a large data set from both purebred and crossbred herds.

In this study, we combined vaginal, uterine, and rectal prolapses into a single trait for analysis instead of analyzing them separately. Several studies in the literature applied the same approach due to the challenging determination of the subcategories. For instance, Supakorn et al. (2019) combined vaginal and rectal prolapse and Bhatia et al. (2023) combined vaginal and uterine prolapse. For sow removal reasons, Arango et al. (2005) investigated the genetic parameters, highlighting the challenge of differentiating between removal reasons related to reproduction and diseases, as some diseases directly affect the reproductive capacity of the sow. They also pointed out that treating each removal reason as a separate trait leads to disregarding information from other reasons. Therefore, they analyzed the reasons for sow removal by grouping the various causes into three groups and performed a multiple-trait analysis, leading to genetic correlation estimates larger than 0.90.

Several studies have reported prolapse incidences by parity and further investigated the effect of parity (Ross, 2019; Supakorn et al., 2019; Bhatia et al., 2023). Ross (2019) found slightly higher prolapse incidence at higher parities. Supakorn et al. (2019) found a higher incidence rate (2%) in parity 1 than in the later parities (0.9%), but this contrast was not significant. Bhatia et al. (2023) observed low incidence rates in parities 1 and > 6 and decided to only use parity 2 to 6 for the analysis. The prolapse incidence rate by parity seems to be population-dependent and might relate to the environmental conditions and management practices at the farm level. In this study, we observed a higher incidence rate in parity 2 than in parity 1 for both purebred and crossbred sows. However, the within-parity incidence rate was lower in parity 2 than in parity 1. Overall, crossbred sows have higher incidence rate than purebred sows. The higher incidence rates in crossbred sows compared to purebred sows may be attributed to differences in the challenges faced by the production systems. Instead of using prolapse events recorded at later parities, we used the prolapse event recorded at parity 2 for sows older than parity 2 and prolapse event recorded at parity 1 for sows at their first parity. Defining the trait at later parities would result in strong censoring, as not all sows reach these parities. Additionally, data collection would take longer. Our aim is to identify an early indicator trait, as a successful early parity performance lays the foundation for lifetime productivity.

Genetic parameter estimation for a binary trait is challenging when the incidence rate is low; this can lead to more significant numerical problems (Misztal et al., 1989; Mäntysaari et al., 1991; Misztal, 2008). With average incidence rates of 0.35% and 0.65% for uterine and rectal prolapses, respectively, Supakorn et al. (2019) reported that modeling the two traits separately failed to converge. With rectal and uterine prolapses combined, their linear model converged with a heritability estimate of 0.03 ± 0.001. However, their threshold model produced a near-zero heritability (0.003 ± 0.003) after 100,000 iterations. Bhatia et al. (2023) avoided the challenge of estimating genetic correlations with a bivariate threshold model by using a bivariate linear model. Small numbers of subclasses without an observed case (e.g., a prolapse) led to numerical problems in the approximation of probability density integrals. With low incidence rates, a large data set is required for genetic analysis.

When using the linear model, the heritability estimate is a function of the incidence rate and the heritability can be transformed to the underlying scale (Hidalgo et al., 2024). Mäntysaari et al. (1991) simulated two correlated traits with incidence rates of 0.05, 0.15, and 0.25 and compared the genetic parameter estimates between linear and threshold models. When incidence rate is high (0.25), the transformation of heritability from observed scale to underlying scale usually works well, but the threshold model became unstable with low incidence (0.05%). The current literature aimed to estimate the heritability using both linear and threshold models but reported a mixed range of heritability for sow prolapse. Supakorn et al. (2019) analyzed removal records of 11,481 Large White sows collected from 2012 to 2017 and reported very low heritability estimates of 0.03 and 0.003 based on linear and threshold models, respectively. They defined the binary trait as sows removed due to prolapse or other reasons, with average prolapse rates of 0.6%–1.6% across the years 2012–2017. Stevens et al. (2021) and Dunkelberger et al. (2022) used the same data source and conducted across-parity genetic analyses for uterine prolapses recorded at the time of removal in purebred sows from two farms in the USA. Stevens et al. (2021) reported heritabilities of 0.15 from a linear model and 0.22 from a threshold model. Dunkelberger et al. (2022) reported heritabilities of 0.13 and 0.22 from a linear model and a threshold model, respectively, with incidence rates of 7.8% and 3.5% for the two farms. With a case-control dataset, Dunkelberger et al. (2022) reduced the full data set to include 986 cases of prolapsed sows and 986 controls and reported heritability estimates of 0.31 and 0.24 for a linear model and a threshold model, respectively.

Later, Bhatia et al. (2023) used a similar data source to continue investigating the genetic basis of prolapse. They performed across-parity and within-parity analyses with data of 20,094 sows collected from 2012 to 2022 in two farms in the USA. For the across-parity analysis, only culled sows were used and the binary trait was defined as culling due to prolapse *versus* culling due to other reasons. Pedigrees were partially incomplete because of some use of pooled semen, so these analyses were genomic-based. The heritability on the underlying scale was estimated as 0.21 (pedigree-based) and as 0.35 (genomic-based). Based on the 10.1% incidence of culling due to prolapse, they translated these estimates to the observed scale as 0.07 and 0.12, respectively. For the within-parity analysis, the binary trait was redefined as culling in that parity due to prolapse, *versus* no culling or culling due to other reasons. The estimated heritabilities on the underlying scale ranged from 0.11 to 0.27 (pedigree-based) and from 0.15 to 0.41 (genomic-based). The genetic correlations between parity 2 *versus* parities 3, 4, 5, and 6 were 0.71, 0.54, 0.50, and 0.45, respectively. They concluded that these moderate to high genetic correlations between parities suggest that susceptibility of prolapse has a similar genetic basis across parities.

The threshold model, initially proposed to evaluate categorical traits for sire models (Gianola and Foulley, 1983), was later extended to animal models (Varona et al., 1999a; and b). While the sire model assumes similar merit among all dams, the animal model allows for the individual assessment of each dam, potentially improving evaluation accuracy. Ramirez-Valverde et al. (2001) evaluated accuracies on beef cattle calving difficulty among a combination of threshold and linear, sire and animal, univariate and bivariate models. Although the threshold animal model outperformed other models for sires with low accuracy, the differences between the models were negligible or non-existent for sires with high accuracy. The accuracy of evaluation for individual animals based on their information when the incidence is low is also low. Therefore, for prolapse evaluation, the linear sire model may be almost as accurate as the threshold animal model. Numerous studies concluded that threshold models showed no apparent advantage over linear models (Weller et al., 1988; Hagger and Hofer, 1989; Matos et al., 1997; Varona et al., 1999b), that linear and threshold models generally yield similar results in terms of strongly correlated estimated breeding values (Hidalgo et al., 2022; Hidalgo et al., 2024), and that genetic correlation estimates were not much affected by the loss of information due to the discreteness of the data (Gianola, 1981; Mäntysaari et al., 1991). In our study, using threshold animal models led to a similar conclusion compared to linear animal model. Therefore, we only report results from the linear animal model on the observed scale. We also performed a linear sire model that yielded similar estimates of heritability with correlation for estimated breeding values of 0.96 for sires.

A trait such as prolapse (with a heritability estimate that is decidedly lower than 0.3 and that is sensitive to the incidence rate and to the volume of data analyzed) is influenced by non-genetic factors much more than by genetic ones. Ross (2019) summarized multiple factors on the farm level and the individual sow level and identified four potential risk factors of prolapse incidence: water treatment and bump feeding strategies on the farm level, and perineal score and body condition. Therefore, reducing prolapse incidence rate will have to rely both on genetic improvement and on improved animal management, and the latter is very likely to be more effective.

With studies reporting mixed ranges of heritability due to the sensitivity and challenges associated with data and model selection in prolapse, our study highlights the importance of robust genetic parameter estimations using large datasets recorded across different environmental conditions and geographical regions. Crucially, our data includes not only nucleus purebred sow herds but also commercial crossbred sow herds, offering a more realistic representation of the industry. Our study is the first to exploit such wide-ranging data for estimating genetic parameters, setting the stage for future research.

## 5 Conclusion

Genetic parameter estimation for pelvic organ prolapse in sows requires large datasets with a high incidence rate across widespread environmental conditions. The heritability is higher in crossbred sows than in purebreds, and it is the incidence rate in commercial crossbred herds that is of primary interest; hence crossbred data are more valuable, but they are more difficult to obtain for proper genetic analysis. Because the heritability of this trait is low, reduction of the prolapse incidence rate will have to rely on genetic improvement and on improved animal management, and the latter is likely to be more effective.

## Data Availability

The data analyzed in this study is subject to the following licenses/restrictions: The datasets generated and analysed in this study are from the Genus PIC breeding program and not publicly available. Requests to access these datasets should be directed to C-YC, ching-yi.chen@genusplc.com.
